# The Eye during Anæsthesia

**Published:** 1908-12-12

**Authors:** 


### Anæsthetics.

### THE EYE DURING ANÆSTHESIA.

Whilst the depth of anæsthesia can best be gauged by the amplitude and regularity of the thoracic movements, it is also possible to obtain useful information from the eye, both from the size of the pupil and its light reflexes and the degree of sensitiveness to touch of the conjunctiva and cornea, as measured by the lid-reflex. It is therefore most necessary to note the size of the pupils and their reactions before the induction of anæsthesia, so as not subsequently to be misled by such factors as the previous administration of drugs, or organic lesions affecting the pupil movements.

During the first stage of anæsthesia nothing can be learnt from the pupil. The patient should be told to keep the eyes closed, and should not be disturbed either by drawing back an eyelid or by touching the conjunctiva or cornea. It is only when the administration has advanced well into the second stage (light anæsthesia) that information can be gained. The resistance of the eyelid on being raised will now probably be absent.

The pupil is then appreciably smaller, and contracts readily to light, enlarging again if the lid be kept raised. It is, however, not always found contracted, and occasionally in adults, and more frequently in children, the pupil will remain dilated till the end of this stage, or even till surgical anæsthesia is established. This condition is frequently associated with a stomach that is not empty. The stomach may contain either the remains of a partially digested meal or the mucus (with or without an admixture of bile) that is so often met with in nervous adults, in patients kept too long a time without food, and in children suffering from adenoids with an associated post-nasal catarrh. Such a stomach may empty itself unaided before the third stage of anæsthesia is reached, or if the condition is diagnosed, as it frequently can be, the process may be initiated by stimulating the fauces with a finger or a sponge.

It should be mentioned that a very small pupil accompanied by quiet and shallow breathing is sometimes associated with this condition of the stomach during the induction of anæsthesia with chloroform. During the second stage, touching the conjunctiva lightly may still evoke a faint response, while a marked lid-reflex will be produced by touching the cornea.

As anæsthesia advances to the third stage (true surgical anæsthesia) the pupil becomes smaller, till under chloroform it averages 2½ mm. to 3 mm., and under ether 4 mm. in diameter; that is, assuming that the induction is proceeding normally, that there is no partial asphyxiation from whatever cause, nor any disturbance of the patient that may give rise to reflex dilatation. The lid-reflex will now be found absent on touching the conjunctiva, but the cornea may still give a slight response. It is at this stage in the majority of cases that the operation can be commenced without eliciting any reflex movements. Until recent years it was more often the custom not to begin the operation until the corneal reflex had been abolished, but it is now recognised that the majority of operations do not require such a depth of anæsthesia. This, of course, depends largely on the patient, and in some cases relaxation can only be maintained by increasing the anæsthesia until the lid-reflex is no longer obtained.

Various causes may give rise to an alteration in size of the pupil during the operation. Too little anæsthetic is evidenced by a gradually dilating pupil with accentuation of the "lid-reflex." On the other hand, a loss of corneal lid-reflex followed by a dilating pupil indicates that the anæsthesia is becoming too deep.

Dilatation due to reflex disturbance from the site of operation is often seen, and is especially common during operations in the neck, *e.g.* for glands, and in other regions when some extra stimulus takes place. In the case of operations in the neck, this dilatation is frequently permanent throughout the operation, and the anæsthetist has to be guided by other signs at his disposal. But when it arises from the extra stimulus, especially when the dilatation continues, it indicates a certain degree of surgical shock, and relatively too deep an anæsthesia, and the anæsthetic should be temporarily withheld.

The minutely contracted or "pin-point" pupil is far more frequently met with under chloroform than ether, and is a sign of an increasingly light anæsthesia, which may suffice in many cases, but is liable, unless care be taken, to end in dilatation of the pupil and subsequent vomiting. This latter condition is almost always accompanied by shallow respiration, and the difficulty of the moment is to get the patient to breathe sufficiently deeply to inhale enough vapour to keep him quietly anæthetised. The remedy with ether is simply to increase the percentage of the vapour, but with chloroform this is frequently insufficient, and the respiratory action must be stimulated by briskly rubbing the face or by adding ether to the chloroform mask, or by changing to ether entirely for the time.

### Coins in the Œsophagus.

It is a remarkable fact that coins, whatever their size, if they stick in the œsophagus, always do so just behind the level of the upper notch of the sternum. One might expect that a smaller coin would have to travel farther down than a larger one, but this is not the case, except in so far as a coin may be so small as not to stick at all, or so large as not to be able to get in. If the coin sticks, this always takes place at the level of the upper notch of the sternum, and in nearly every case with the flat surfaces anterior and posterior. This has been confirmed again and again with the *x*-rays.

